# The incretin effect in type 2 diabetes in a Sub-Saharan African population

**DOI:** 10.1186/s40842-024-00178-5

**Published:** 2024-07-25

**Authors:** Signe Tellerup Nielsen, Belinda Kweka, George Praygod, Suzanne Filteau, Mette Frahm Olsen, Henrik Friis, Daniel Faurholt-Jepsen, Rikke Krogh-Madsen

**Affiliations:** 1 Department of Anesthesia Surgery and Intensive Care, Copenhagen University Hospital - Herlev, Herlev, Denmark; 2 Department of Anesthesiology and Intensive Care, Copenhagen University Hospital - Hvidovre, Hvidovre, Denmark; 3 National Institute for Medical Research, Mwanza, Tanzania; 4 Faculty of Epidemiology and Population Health, London School of Hygiene and Tropical Medicine, London, UK; 5 Department of Infectious Diseases, Copenhagen University Hospital - Rigshospitalet, Copenhagen, Denmark; 6 Department of Nutrition, Exercise and Sports, University of Copenhagen, Copenhagen, Denmark; 7 Centre for Physical Activity Research, Rigshospitalet, University of Copenhagen, Copenhagen, Denmark; 8 Department of Infectious Diseases, Copenhagen University Hospital - Hvidovre, Hvidovre, Denmark

**Keywords:** Incretin effect, Glucagon-like peptide 1 (GLP-1), Gastric inhibitory peptide (GIP), Type 2 diabetes, Sub-Saharan Africa

## Abstract

**Aim:**

Type 2 diabetes is increasing in Sub-Saharan Africa, but the pathophysiology in this population is poorly investigated. In Western populations, the incretin effect is reduced in type 2 diabetes, leading to lowered insulin secretion. The aim of this study was to investigate the incretin effect in a group of Sub-Saharan Africans with type 2 diabetes.

**Methods:**

Twenty adults diagnosed with type 2 diabetes, based on either an oral glucose tolerance test (*n* = 10) or on glycated hemoglobin A1c (*n* = 10), and 10 non-diabetic controls were included in an interventional study in Tanzania. We investigated the incretin effect as the difference between the plasma insulin area under the curve during an oral glucose tolerance test and that obtained during an intravenous glucose infusion. Differences between diabetes groups were analyzed by Kruskal–Wallis one-way analysis of variance.

**Results:**

The incretin effect did not differ between groups (*p* = 0.45), and there was no difference in plasma concentrations of the incretin hormones during the OGTT.

**Conclusion:**

A reduced incretin effect appears not to contribute to hyperglycemia in type 2 diabetes in this Tanzanian population. More research is needed to explain the diabetes phenotype often seen in Sub-Saharan Africa.

**Trial registration:**

Clinicaltrials.gov: NCT03106480, date of registration: 04/10/2017.

## Introduction

Type 2 diabetes (T2D) is increasing worldwide, including in Asia and Africa [1]. In Western populations, the pathophysiology and phenotype of T2D are well described and consist of obesity, physical inactivity and metabolic changes including peripheral insulin resistance, low grade systemic inflammation and reduced incretin effect [2–4]. The incretin effect refers to the greater release of insulin to the circulation after oral glucose ingestion than after intravenous glucose infusion producing the same blood glucose profile. Glucagon-like peptide 1 (GLP-1) and gastric inhibitory peptide (GIP) are incretin hormones responsible for the incretin effect [2, 5]. The importance of the incretin effect for the maintenance of glucose homoeostasis is well established [6] and is responsible for up to 70% of the total insulin secretion related to a glucose stimulus in healthy persons [2]. Thus, incretin hormone-based therapies are widely used in the treatment of T2D.

The T2D diagnosis can be based on elevated fasting blood glucose, elevated blood glucose after a 2-hour oral glucose tolerance test (OGTT), or an elevated glycated hemoglobin A1c (HbA1c) [1]. The phenotype of T2D in Sub-Saharan African (SSA) and Asian populations differs from the one of Western populations regarding obesity and physical inactivity [7, 8] There seems to be a higher frequency of severe insulin-deficient diabetes mellitus, younger age and lower body mass index (BMI) at diagnosis, lower β-cell function, and lower insulin resistance among Indian and Chinese people compared with European people [9]. In a large Tanzanian cohort we have previously shown that HbA1c and OGTT identified different participants as having diabetes or prediabetes [8]. The phenotype of T2D in SSA is suggested to be linked with beta-cell secretory dysfunction rather than insulin resistance [10]. This differs from data reported from the Western world where progression of T2D is described with an early development of insulin resistance, fully compensated by an increase in insulin secretion. This balance maintains normoglycemia, but still reflects some degree of beta-cell dysfunction. When the increase in insulin resistance coincides with a progressive pancreatic beta-cell secretory dysfunction (relative insulin deficiency), T2D is manifested [11].

In this study, we therefore aimed to determine whether adult Tanzanians with T2D (diagnosed using either 2 h OGTT or HbA1c) have a compromised incretin effect, and if so, whether this could contribute to the T2D pathophysiology in this population.

## Methods

Thirty adult participants were recruited from the Chronic Infections, Co-morbidities, and Diabetes in Africa (CICADA) cohort in Mwanza, Tanzania representing a mixed urban population in SSA [8]. Inclusion criteria for the present interventional study, were age ≥ 18 years, negative HIV status, diabetic status investigated as a part of the CICADA protocol and eligibility in the study period. Ten of the participants were previously diagnosed with T2D by a two-hour OGTT with a plasma-glucose level > 11 mmol/l (T2D-OGTT) and 10 participants were previously diagnosed with T2D by a HbA1c > 48 mmol/mol but had a two-hour OGTT plasma-glucose level < 11.1 mmol/l (T2D-HbA1c). The remaining 10 participants were recruited as non-diabetic controls (NDC), defined as HbA1c < 48 mmol/mol and two-hour OGTT plasma-glucose level < 11.1 mmol/l [1]. Exclusion criteria were positive HIV status, insulin treatment and pancreatic disease. The NDC group was matched to the T2D-OGTT group on age, sex, and body mass index (BMI). Oral anti-diabetes treatment was paused 48 h prior to the first study day.

The study was performed on two separate study days in an experimental design. On the first study day, a 3-hour OGTT (75 g glucose diluted in 300 ml water) was conducted. On the second study day a 3-hour corresponding intravenous glucose infusion (IVGI) (using 10% glucose) that mimicked the glucose profile obtained during the OGTT was performed.

Blood samples were collected and handled identically on the two study days. Blood glucose was measured every five minutes during the first 60 min and every ten minutes during the last 120 min (Hemocue, Sweden).

Blood for measurement of insulin was collected at baseline and subsequently every 15 min for the first hour and every 30 min for the remaining two hours. Tubes were kept upright for 20–40 min at room temperature, then centrifuged at 3500 rpm and 4 °C for 15 min, following which the supernatant was stored at -80 °C until analysis. Insulin was measured using a sandwich electrochemiluminescence immunoassay (Roche, Switzerland).

Blood for measurement of GLP-1 and GIP was collected in chilled EDTA tubes at baseline, and at 15-, 20-, 60-, and 180-min. Tubes were kept on ice and centrifuged immediately at 3500 rpm and 4 °C for 15 min, following which the supernatant was stored at -80 °C until analysis. Plasma GLP-1 and GIP were measured using enzyme-linked immunosorbent assay (Millipore and Meso Scale Discovery, USA).

The incretin effect was calculated as [12]:

- Total area under the curve (tAUC)insulin_OGTT_ – tAUCinsulin_IVGI_) / tAUCinsulin_OGTT_.

As indices of insulin resistance/sensitivity, the following parameters were calculated [13]:

- Matsuda index: 10,000/√ ((baseline glucose *mean glucose) * (baseline insulin * mean insulin)). Insulin measured in microlU/l and glucose in mg/dl.
- Homeostatic model assessment for insulin resistance (HOMA-IR): (fasting glucose * fasting insulin) / 22.5. Insulin measured in microIU/ml and glucose in mmol/l.

As indices of pancreatic β-cell insulin secretory function, the following parameters were calculated [13]:

- Homeostatic model assessment for beta cell function (HOMA-beta): (20 * fasting insulin) / (fasting glucose-3.5). Insulin measured in microIU/ml and glucose in mmol/l.
- Insulinogenic index (IGI): (30 min insulin - fasting insulin) / (30 min glucose - fasting glucose). Insulin measured in microIU/ml and glucose in mg/dl.
- Disposition index (DI): IGI/HOMA-IR.

### Data analysis

Data are presented as medians with interquartile range unless otherwise indicated. Comparisons between groups were based on the Kruskal–Wallis one-way analysis of variance. tAUC was calculated using the trapezoidal rule. Eight missing data points were imputed using the average of the value recorded one time point before and one time point after the missing value. *P* < 0.05 was considered statistically significant. All analyses were performed using STATA version 17.

## Results

The baseline characteristics including indices of insulin resistance/sensitivity are summarized by groups in Table 1. Due to missing data only *n* = 9 in the T2D-OGTT group were included in the analyses. Two of the IVGIs in the T2D-HbA1c group failed, resulting in *n* = 8 in the calculation of the incretin effect in this group. There was no difference between glucose levels on the two experimental days (OGTT/IVGI), indicating that the method was well performed (Table 2). The median incretin effect was 0.47 (0.24–0.52) in the T2D-OGTT group, 0.61 (0.26–0.74) in the T2D-HbA1c group, and 0.50 (0.41–0.64) in NDC group, with no differences between groups (Table 2). There were no differences in the OGTT induced incretin secretion between groups (Table 2).

**Table 1:** Baseline characteristic and indices of insulin resistance/sensitivity of 30 Tanzanian adults stratified by T2D status

	Non-diabetes control *n* = 10		T2D from HbA1c *n* = 10		T2D from OGTT *n* = 10	
	Mean	SD	Mean	SD	Mean	SD
Age (years)	47.2	± 7.5	37.3	± 6.1	48.2	± 7.7
Males (%)	60		10		60	
Weight (kg)	64.0	± 10.8	69.8	± 7.0	66.9	± 10.0
Body mass index (kg/m^2^)	23.1	± 3.4	27.0	± 3.0	24.0	± 4.0
2 h OGTT glucose (mmol/l)	7.5	± 1.2	7.3	± 0.5	13.1	± 4.5
HbA1c (mmol/mol)	38.5	± 4.6	53.9	± 22.1	61.2	± 31.4
Anti-diabetes treatment (%)	0		0		40	
	Median	IQR	Median	IQR	Median	IQR
Matsuda index	6.5	4.2–12.8	5.6	2.6–7.2	7.9	5.0-10.2
HOMA-IR	1.4	0.5–2.2	1.6	1.2-3.0	1.2	0.7–2.7
HOMA-beta	21.7	12.6–42.2	37.6	24.3–76.7	15.5	9.1–22.7
Insulinogenic index	73.1	50.5-127.1	220.2	158.1-378.2	32.2	22.1–81.4
Disposition index	54.3	29.1-112.9	178.4	97.5–317.0	23.1	5.6–59.7

Data are presented as mean ± SD and as median with IQR (when not normally distributed), sex and treatment in percent. HbA1c at inclusion in the CICADA cohort

*Abbreviations: T2D* Type 2 diabetes, *OGTT* Oral glucose tolerance test, *HOMA-beta *Homeostatic model assessment for beta cell function, *HOMA-IR *Homeostatic model assessment for insulin resistance

**Table 2:** Incretin effect and incretin hormone concentrations among 30 Tanzanian adults stratified by diabetes status

		Non diabetes control		T2D from HbA1c		T2D from OGTT		
	Modality	Median	IQR	Median	IQR	Median	IQR	*P*
Incretin effect	OGTT/IVGI	0.50	0.41–0.64	0.61	0.26–0.74	0.47	0.24–0.52	0.45
GLP-1, tAUC	OGTT	1,055	712-1,599	1,085	880-1,118	1,082	972-1,711	0.87
GLP-1, tAUC	IVGI	435	313–535	357	326–384	686	413–942	0.07
GIP, tAUC	OGTT	37,996	26,567 − 40,586	36,668	33,888 − 46,386	41,502	26,393 − 53,141	0.79
GIP, tAUC	IVGI	8,137	7,166 − 21,213	4,580	3,473-7,512	12,804	8,307 − 14,079	0.06
Glucose, tAUC	OGTT	1,414	1,240-1,593	1,378	1,272-1,452	1,728	1,596-2,852	
Glucose, tAUC	IVGI	1,466	1,324-1,537	1,421	1,329-1,466	1,743	1,630-3,062	
Insulin, tAUC	OGTT	43,251	28,433 − 53,433	54,871	42,847 − 77,333	23,352	16,492 − 41,101	
Insulin, tAUC	IVGI	16,486	15,332 − 30,167	23,537	19,441 − 39,660	11,041	8,631 − 25,331	

Data are median, interquartile ranges (IQR) and p-values from Kruskal–Wallis one-way analysis of variance. Data on glucose and insulin during OGTT/IVGI is presented for informational purposes.

*Abbreviations: T2D* Type 2 diabetes, tAUC Total area under the curve, OGTT Oral glucose tolerance test, IVGI Intravenous glucose infusion

## Discussion

We found no difference in the incretin effect between non-diabetic controls and participants with T2D, irrespectively of how the T2D was diagnosed. A possible explanation could be that the decreased incretin effect in T2D may occur after the diabetes diagnosis is established and, therefore, is a consequence of the diabetic state [14]. A reduced expression of GIP receptors and/or a reduction in beta-cell mass and functional insulin secretory capacity are suggested as possible underlying mechanisms, maybe triggered by glucotoxicity/hyperglycemia [15]. Thus, the observed normal incretin effect in participants with T2D in our study could be due to their relatively young age so they may be at an early stage in the progression of their diabetes disease.

We found no differences between diabetes groups in incretin hormone concentrations during the OGTT, which is supported by a meta-analysis suggesting that there are no differences in OGTT-induced secretion of the incretins GIP and GLP-1 between healthy individuals and individuals with T2D [16].

The present study is limited by the relatively small sample size. However, the sample size was based on a power calculation, and previous studies have shown differences in incretin effect in similarly small study populations [17]. The number of missing data was low. Still, the dataset is rather small and missing data can potentially influence the results.

## Conclusion

A reduced incretin effect did not seem to contribute to hyperglycemia in T2D in this SSA population. Since incretin hormone-based therapies are becoming more and more common in the treatment of T2D, more and larger studies are warranted before establishing incretin-based therapies in SSA populations.

## Data Availability

Supporting data and materials are available on request. Please contact the corresponding author.

## Abbreviations

BMI: Body mass index
DI: Disposition index
EDTA: Ethylen diamin tetra acetid acid
GIP: Gastric inhibitory peptide
GLP-1: Glucagon-like peptide 1
HbA1c: Glycated hemoglobin A1c
HOMA-beta: Homeostatic model assessment for beta cell function
HOMA-IR: Homeostatic model assessment for insulin resistance
IGI: Insulinogenic index
IVGI: Intravenous glucose infusion
NDC: non-diabetic controls
OGTT: Oral glucose tolerance test
SSA: Sub-Saharan Africa
tAUC: Total area under the curve
T2D: Type 2 diabetes
T2D-HbA1c: HbA1c above 48 mmol/mol and a two-hour OGTT plasma-glucose level less than 11.1 mmol/l
T2D-OGTT: T2D diagnosed by a two-hour OGTT plasma-glucose level above 11 mmol/l
